# Paradoxical Association of C-Reactive Protein with Endothelial Function in Rheumatoid Arthritis

**DOI:** 10.1371/journal.pone.0010242

**Published:** 2010-04-27

**Authors:** Michael V. Holmes, Benyu Jiang, Karen McNeill, Melinda Wong, Stephen P. Oakley, Bruce Kirkham, Phil J. Chowienczyk

**Affiliations:** 1 King's College London, British Heart Foundation Centre, Department of Clinical Pharmacology, St. Thomas' Hospital, London, United Kingdom; 2 Department of Rheumatology, Guy's and St. Thomas' National Health Service Foundation Trust, London, United Kingdom; 3 National Institute for Health Research, Guy's and St. Thomas' National Health Service Foundation Trust, King's College London Biomedical Research Centre, London, United Kingdom; University of Illinois at Chicago, United States of America

## Abstract

**Background:**

Within the general population, levels of C-reactive protein (CRP) are positively associated with atherosclerotic cardiovascular disease (CVD). Whether CRP is causally implicated in atherogenesis or is the results of atherosclerosis is disputed. A role of CRP to protect endothelium-derived nitric oxide (EDNO) has been suggested. We examined the association of CRP with EDNO-dependent vasomotor function and subclinical measures of atherosclerosis and arteriosclerosis in patients with raised CRP resulting from rheumatoid arthritis (RA).

**Methodology/Principal Findings:**

Patients with RA (n = 59) and healthy control subjects (n = 123), underwent measures of high sensitivity CRP, flow-mediated dilation (FMD, dependent on EDNO), intima-media thickness (IMT, a measure of subclinical atherosclerosis) and aortic pulse wave velocity (PWV, a measure of arteriosclerosis). IMT and PWV were elevated in patients with RA compared to controls but FMD was similar in the two groups. In patients with RA, IMT and PWV were not correlated with CRP but FMD was positively independently correlated with CRP (P<0.01).

**Conclusions/Significance:**

These findings argue against a causal role of CRP in atherogenesis and are consistent with a protective effect of CRP on EDNO bioavailability.

## Introduction

C-reactive protein (CRP), a biomarker of systemic inflammation, independently associates with endothelial dysfunction, subclinical atherosclerosis and arteriosclerosis[1], [2] and clinical manifestations of atherosclerosis in the general population[3], [4]. CRP has been advocated for use in prediction of cardiovascular disease (CVD) risk[5], [6]. It has been suggested that systemic inflammation leads to endothelial activation and dysfunction and that CRP itself may be a causal factor in atherogenesis[7], [8]. It is notable that reduction of CRP by a statin in those with low/normal LDL is associated with a marked reduction in CVD events[9]. However, it is also possible that CRP is produced by inflammation within atherosclerotic plaque and therefore associates with atherosclerosis by reverse causality[10]. It is thus important to clarify, whether when raised levels of CRP arise from a non-vascular cause (i.e. a stimulus for CRP other than atherosclerosis), CRP retains its association with CVD. This would validate the potential use of CRP for risk assessment irrespective of co-morbidity and/or the cause of raised CRP, and would add weight to the putative causal role of CRP in CVD.

Rheumatoid arthritis (RA) is a chronic inflammatory condition associated with subclinical vascular disease and a greater prevalence of CVD than can be accounted for by traditional risk factors alone; compared to controls, the relative risk of myocardial infarction in RA is doubled[11]. CRP in RA is usually increased into the range associated with increased CVD risk[12] raising the possibility that CRP is responsible for accelerated atherogenesis in RA. The purpose of the present study was to examine the association of CRP with vascular function and structure in RA to determine if CRP is likely to be a useful marker of sub-clinical atherosclerosis and increased CVD risk in RA and whether CRP retains its association with subclinical CVD in a systemic inflammatory condition where a major stimulus to production of CRP arises from inflammation outwith atherosclerotic plaques.

We examined endothelial function (by flow mediated dilation, FMD, a measure of EDNO), intima-media thickness (IMT, a measure of subclinical atherosclerosis) and large artery stiffness by pulse wave velocity (PWV, a measure of arteriosclerosis). These measures of subclinical CVD are all strongly predictive of CVD[13]–[26].

## Methods

Patients with RA (n = 65, complete data subsequently available in n = 59) fulfilling the American College of Rheumatology classification criteria[27] were recruited from the rheumatology clinics of Guy's and St Thomas' NHS Foundation Trust Hospital. Patients had not previously been treated with biological agents (e.g. TNF-α antagonists). Patients were excluded if they had evidence of: inter-current infections; coronary heart disease; cerebrovascular disease; peripheral disease; diabetes mellitus, or if they were receiving HMG-coA reductase inhibitors (statins) or aspirin. Healthy age matched controls (n = 123) recruited from the local community were studied contemporaneously. The study was approved by the Guy's and St Thomas' NHS Foundation Trust Research Ethics Committee. All subjects gave written informed consent.

### Protocol

Subjects attended in the morning, having refrained from caffeine and alcohol for 12 hours, and vascular measurements were made in a quiet temperature controlled laboratory after a standardised light breakfast. Blood pressure was measured seated using an automated oscillometric device (Omron HEM 705, Omron, Japan). Blood for biochemistry was obtained after an overnight fast on a day prior to (and within 8 weeks) of the vascular measurements. CRP was measured using high-sensitivity turbidimetric immunoassay (WAKO Chemicals) on a Cobas Mira Analyser (Roche Diagnostics).

### Endothelial function

Endothelial function was assessed by measuring FMD of the brachial artery[28] according to current guidelines. High resolution ultrasound (Aspen, Siemens, Germany with 7 MHz linear array transducer, positioned by a stereotactic manipulator) was used to scan the brachial artery in a longitudinal section 2 to 15 cm above the elbow. After optimal positioning of the transducer a baseline scan was recorded. Increased flow was then induced by inflation of a pneumatic tourniquet placed around the forearm (distal to the arterial segment being scanned) to a pressure of 250 mmHg for 5 min, followed by release. A second scan commenced 10 sec before release of the cuff and continued for 3 min after cuff deflation. After 10 min to allow vessel recovery, another resting scan was taken. Sublingual nitroglycerine (NTG, 25 µg) was then administered, and a final scan performed 3 to 4 min later. Images were coded and recorded on VHS videotape, then digitized for subsequent blinded analysis using automated edge detection software (Brachial Analyser, Medical Imaging Applications, LCC, Iowa, USA). FMD was expressed as the percentage increase in brachial artery diameter from baseline to maximal dilation which occurred 30 to 90 sec after release of the cuff. Dilation to NTG was expressed as the percentage increase in brachial artery diameter from baseline to maximal dilation after NTG.

### Common carotid intima-media thickness

High resolution ultrasound (as for FMD) was used to image the left and right common carotid arteries. IMT was measured from digitized images obtained in diastole of the near and far walls of both common carotid arteries 1–2 cm proximal to the flow divider. This method provides a robust measure of IMT with reproducibility higher than that in other segments[19].

### Carotid-femoral pulse wave velocity

PWV was measured over the carotid-femoral portion of the arterial tree by ECG referenced sequential carotid and femoral tonometry using the SphygmoCor system (Atcor, Australia). Transit time was measured from the foot to foot delay between the carotid and femoral pulse waveforms. Carotid-femoral distance was estimated from the distance between the suprasternal notch and the femoral artery at the site of transducer placement. All measurements were made by an experienced observer and readings which did not conform to the internal quality checks provided by the SphgmoCor software were rejected. Measurements were made in triplicate with mean values used for analysis.

### Statistical analyses

There were 6 subjects with incomplete data in the RA group (due to errors in laboratory handling of samples) and the analysis was restricted to n = 59 in whom complete data were available. The relation between vascular measures and risk factors other than CRP was examined (in each group separately and in the combined groups) by univariate regression analysis. Vascular measures were compared in the RA and control groups by analysis of covariance, incorporating potential confounding values identified on the univariate analysis. The relationship between vascular measures and CRP within the RA group was examined by regression analysis with CRP treated both as a continuous variable and with CRP categorised into tertiles as an ordinal variable. All potential confounding variables indentified on univariate analysis were again incorporated as co-variates. Analyses were performed using SPSS for Windows version 17 and P<0.05 was considered as evidence against the null hypothesis.

## Results

### Subject characteristics

Subject characteristics (RA n = 59, healthy controls n = 123) are shown in Table 1. Within the RA group, compared to the control group, there were fewer men, mean values of BMI were lower, blood pressure higher and HDL-cholesterol lower (table 1). 20% of RA subjects were current smokers. CRP was higher in RA patients (median 9.10 mg/l, IQR 5.00–17.30) compared to controls (median 0.30 mg/l, IQR 0.04–1.22, P<0.0001).

**Table 1:** Characteristics of rheumatoid arthritis patients and healthy controls.

	Controls (n = 123)	Rheumatoid arthritis (n = 59)		
		CRP ≤5 mg/L (n = 20)	CRP 5 to 13 mg/L (n = 19)	CRP >13 mg/L (n = 20)
Age (mean years ± SD)	48.97±9.87	50.85±8.63	51.32±10.54	49.20±14.04
Sex (females, %)	73 (59.35)	15 (75.00)	17 (89.47)	16 (80.00)
BMI (mean kg/m^2^ ± SD)	27.72±3.90‡	24.12±3.19	26.16±7.28	26.34±6.64
Present smokers (number, %)	0	6 (30.00)	4 (21.05)	2 (10.00)§
Systolic blood pressure (mean mmHg ± SD)	117.59±15.08	122.58±19.02	126.00±14.84	126.53±15.90
Diastolic blood pressure (mean mmHg ± SD)	73.47±8.12	77.59±9.60	77.21±8.78	76.57±8.08
Total cholesterol (mean mmol/l ± SD)	5.40±1.02*	5.30±0.97	5.57±1.02*	4.70±0.82
HDL-cholesterol (mean mmol/l ± SD)	1.51±0.40*	1.44±0.42*	1.37±0.41	1.12±0.23
LDL-cholesterol (mean mmol/l ± SD)	3.36±0.83	3.44±0.85	3.72±0.90	3.06±0.78
TG (mean mmol/l ± SD)	1.28±0.69‡	0.87±0.37	1.04±0.48	1.10±0.49
CRP (median mg/l, IQR)	0.30 (0.04, 1.22)	3.35 (2.40, 5.00)	9.10 (7.20, 10.90)	25.85 (16.65, 39.40)
**Rheumatoid arthritis disease parameters**				
Disease duration (median years ± IQR)	-	9.00 (2.00, 20.00)	12.50 (2.00, 19.50)	9.00 (3.00, 21.00)
Disease Activity Score (mean ± SD)	-	3.90±1.53	5.13±1.19‡	5.78±1.10‡
Health Assessment Questionnaire (mean ± SD)	-	0.93±0.77	1.64±0.94‡	1.78±0.81‡
**Drug therapy** †				
Methotrexate (no, %)	-	10 (50.00)	15 (78.95)	16 (80.00)
Sulphasalazine (no, %)	-	7 (35.00)	10 (52.63)	8 (40.00)
Hydroxychloroquine (no, %)	-	2 (10.00)	6 (31.58)	4 (20.00)
Leflunamide (no, %)	-	2 (10.00)	3 (15.79)	0
Gold (no, %)	-	1 (5.00)	0	0
NSAIDs (including cyclo-oxygenase 2 inhibitors, no, %)	-	1 (5.00)	6 (31.57)	10 (50.00)

BMI: body mass index; HDL: high-density lipoprotein; LDL: low-density lipoprotein; TG: triglycerides; CRP: C-reative protein; NSAIDs: non-steroid anti-inflammatory drugs; SD: standard deviation; IQR: interquartile range.

†No patient with rheumatoid arthritis received azathioprine treatment at the time of enrolment to the study.

*P<0.05 compared to 3^rd^ (>13 mg/L) CRP tertile.

‡P<0.05 compared to 1^st^ (≤5 mg/L) CRP tertile.

§distribution of smoking in RA patients P = 0.29 (Fisher's exact test).

Rheumatoid arthritis patients are grouped into tertile of C-reactive protein.

### Relation of vascular measures to risk factors other than CRP

Within the control group and the combined groups, measures of vascular function and structure were associated with classical risk factors for atherosclerosis, most strongly with age and systolic blood pressure. On multivariate analysis, in the combined groups, FMD was independently negatively associated with age (P<0.01), systolic blood pressure (P<0.05) and tended to be negatively associated with total cholesterol (P<0.1). IMT was independently associated with age (P<0.001) and systolic blood pressure (P<0.01). PWV was independently associated with age (P<0.001) and systolic blood pressure (p<0.001).

### Comparison of vascular measures in RA and control groups

When adjusted for all potential confounding factors found to be correlated with vascular measures on univariate analysis (P<0.05), FMD was not different between RA and control groups (6.22±0.33% vs. 6.55±0.22%, means ±SEM, P = NS). Results were similar when the analysis was restricted to non-smokers (6.30±0.37% vs. 6.60±0.21%, P = NS). IMT was greater in RA compared to control subjects (0.90±0.03 mm vs. 0.82±0.01 mm, P<0.05). PWV was also greater in RA compared to control subject (8.69±0.18 m/s vs. 8.02±0.12 m/s, P<0.01).

### Relation of vascular measures to CRP within the RA group

**Figure 1:**
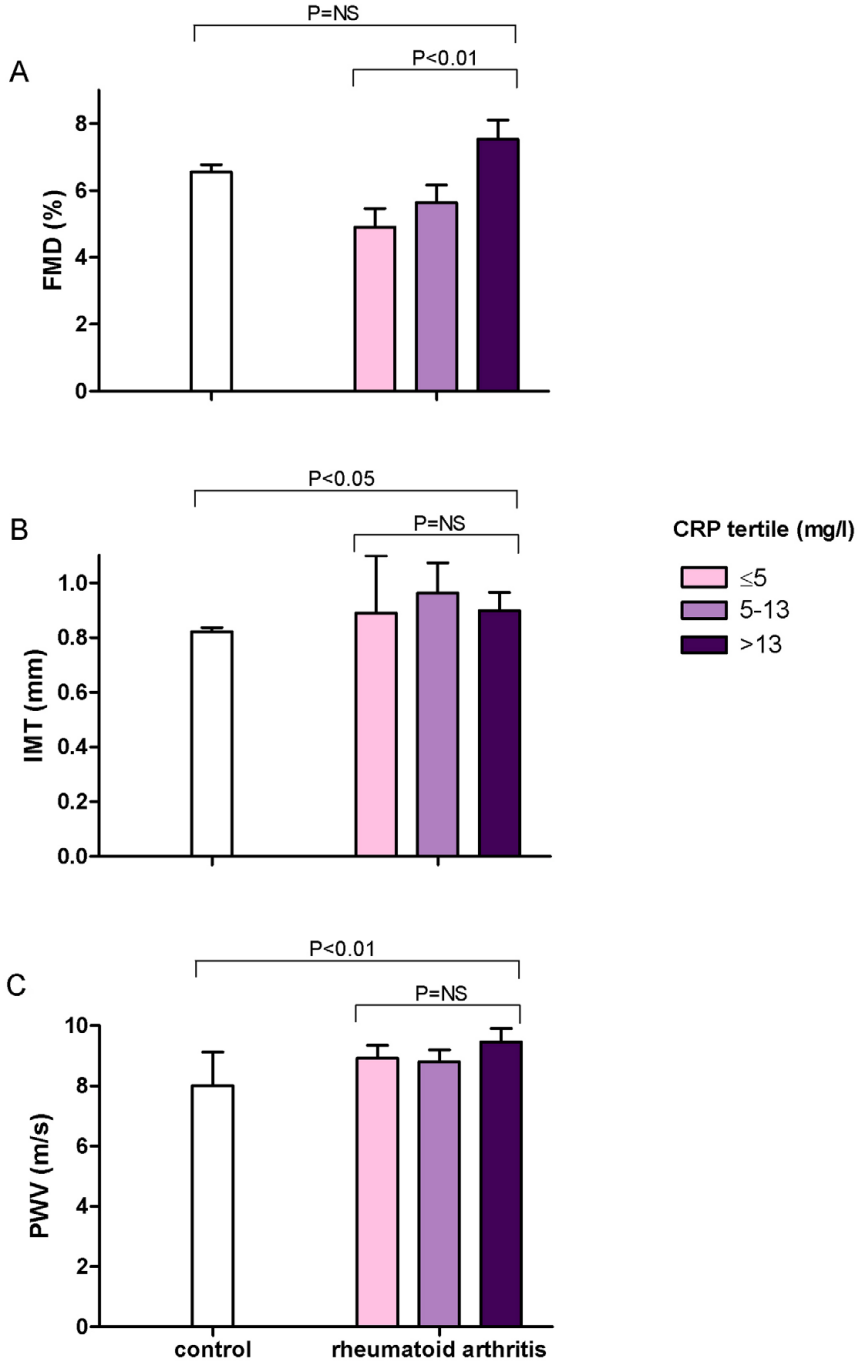
Relationship between tertiles of C-reactive protein in rheumatoid arthritis patients (n = 59) and subclinical markers of coronary heart disease. A) FMD (flow-mediated dilatation), B) IMT (intima medial thickness), C) PWV (pulse wave velocity). FMD/IMT/PWV values are estimated marginal means (± SEM) derived from multiple regression analysis adjusted for potential confounders. Healthy controls (n = 123) are displayed for reference.

Within the RA group, FMD was positively associated with CRP (P<0.01, by multiple regression analysis incorporating age, sex, body mass index, smoking status, systolic blood pressure, total cholesterol and high density lipoprotein, disease activity, disease duration and concurrent drug treatment). FMD also positively correlated with CRP in non-smokers with RA (P<0.05) on multivariate regression analysis. IMT and PWV were not associated with CRP (Figure 1). There was no relationship between vascular measures and disease duration/activity as measured by disease activity score, nor with the health assessment questionnaire score. These findings were similar irrespective of whether the analysis was performed using CRP as a continuous variable or categorized into tertiles.

## Discussion

We devised our study to investigate if raised CRP occurring in a chronic inflammatory disease associates with subclinical CVD. This was for two reasons. Firstly if CRP is to be useful in CVD risk prediction in patients with chronic inflammatory disease, a close association between CRP and CVD would be mandated, and secondly, if CRP is causal in CVD, a raised CRP from whatever cause should associate with CVD. Contrary to what might be expected, in patients with elevated CRP due to chronic disease activity in RA, IMT and PWV, structural measures of early atherosclerosis/arteriosclerosis, were not related to CRP and, intriguingly, the reverse association was found for FMD: higher CRP levels correlated with higher FMD i.e. higher levels of CRP were associated with better, not worse, endothelial function. This association remained robust when potentially confounding factors such as disease duration, activity of disease and concurrent drug treatment were controlled for. As far as we are aware, this is the first study to report such a paradoxical association between FMD and CRP in subjects with a chronic inflammatory condition.

These findings are difficult to reconcile with CRP as a causal factor in CVD. The findings with respect to IMT and PWV are, however, consistent with the association between CRP and CVD being driven by reverse causality (i.e. CVD causing raised CRP). Values of CRP in the mid and upper tertiles of the distribution in patients with RA were greater than those found in subjects with subclinical CVD but without another stimulus to CRP production (such as RA). Thus a lack of association between CRP and CVD would be expected in RA if CRP arising from a stimulus unrelated to atherosclerosis is unrelated to CVD. It is notable that genetic epidemiological studies support such reverse causality[29]–[33].

Reverse causality in the link between CRP and CVD would not explain the apparent paradoxical association of FMD and CRP seen in the present study with better FMD associated with higher levels of CRP. Unlike IMT and PWV which are structural changes associated with early atherosclerosis/arteriosclerosis in the subendothelial arterial wall, FMD is a measure of endothelial cell function. Although generally regarded as a surrogate for generalized endothelial cell function,[34] FMD is mainly determined by the availability of EDNO[35] which, as well as regulating arterial tone, has antiatherogenic functions[36], [37]. In the presence of Reactive Oxygen Species, NO can be deleterious to the endothelium[38], yet FMD measures the vasodilatory (protective) function of EDNO in response to shear stress of the vessel wall[28]. It is notable that previous studies show that pure human CRP has specific, direct effects on vascular function in vitro via increased NO production[39] and that following an acute inflammatory insult, NO-dependent vasodilator function is immediately impaired but returns to normal when CRP rises[39]. Our findings are entirely consistent with such a protective role of CRP on NO availability and hence FMD. They may explain why FMD is not always impaired in subjects with RA compared with healthy control subjects[40]. A protective effect of CRP on NO availability would be expected to abrogate adverse effects of systemic inflammation on aspects of atherogenesis other than those dependent on NO and suggests that these may be partially masked by the increased CRP associated with inflammation. Of note, prospective studies have demonstrated that CRP is associated with cardiovascular end-points in RA[41], [42]. With *prevalent* CVD, CRP may be a by-product of atherosclerosis and its association could result from reverse causation; our study could not demonstrate this as our RA patients had not experienced incident CVD. An alternative explanation, by analogy to NO[38], is that CRP may have pleiotropic roles, some protective and others harmful.

These data are of relevance to CVD prevention through reduction in inflammation. In the general population, targeting individuals with raised CRP for statin therapy reduces CVD events[9] and it has been suggested that these results can be extrapolated to subjects with chronic autoimmune disease such as RA[43]. Our findings suggest that the use of CRP in RA (and perhaps other chronic inflammatory disease) should be further evaluated before being used as a risk marker of CVD.

Our study was limited in several ways. The cross-sectional design does not provide definitive evidence of causality or reverse causality although, as discussed above, the results do strongly argue against a detrimental effect of CRP on all aspects of endothelial dysfunction and atherosclerosis, particularly those involving NO availability. We measured vascular function/structure and CRP only at a single time point. Temporal variation of these properties would, however, tend to obscure the most striking observation in this study: the paradoxical association of FMD with CRP. Our conclusions relate only to subclinical measures of CVD: endothelial vasomotor NO-dependent function, intima-media thickening and arterial stiffening. Although each of these measures is strongly related to clinical CVD they are not the only determinants of CVD events. Results relating to atherosclerosis and CVD mortality in relation to CRP in RA are conflicting[41], [42], [44] and CRP could have a role relating to thrombogenesis independent of atherogenesis[7].

In conclusion, CRP in patients with RA is unrelated to structural measures of subclinical arterial disease and there is a paradoxical association of CRP with improved endothelial function. These observations argue against a causal role of CRP in atherosclerosis. They are consistent with reverse causality in the association of CRP with atherosclerosis and a protective effect of CRP on NO availability. Further studies are warranted to determine if there is a dissociation of CRP with clinical CVD events in chronic inflammatory conditions and interventional studies are required to determine if there are potential protective effects of CRP on aspects of endothelial function.
